# Dr. Beaver's Case of Cynanche Trachealis

**Published:** 1811-11

**Authors:** Robert Beaver

**Affiliations:** Carnarvon, North Wales


### *To the Editors of the Medical and Physical Journal.*

GENTLEMEN,

PHYSICIANS in their writings have not recorded many instances of Cynanche Trachealis in the adult subject.—Notwithstanding my most minute research into medical histories, I have only met with one solitary case, in the person of that justly celebrated, and truly venerable character, General Washington.

The subjoined case has very recently occurred in my practice.

On Sunday, the 28th of April, 1811, about ten o'clock in the evening, a Surgeon and Apothecary in this town was desired to see Mr. Roberts, a printer and bookseller, in his fiftieth year. Apprehending danger, he requested further advice; and at half past ten I visited the patient. The attendants informed me that Mr. R. had been complaining, in a trifling degree, for two or three days, of a hoarseness and slight cough, with some difficulty of respiration; but the symptoms were not deemed of sufficient importance to create any alarm in the mind of the patient or his friends, or to induce them to call in medical aid.

I found him in a half reclined posture, breathing with considerable difficulty.—He seemed very averse to speaking, answered my questions in a peculiar shrill tone of voice, and the sound of inspiration resembled that of air passing through a metallic tube. His countenance expressed the greatest anxiety, and was marked by sudden transitions from a deep red, to death-like paleness. He was harassed with a constant shrill cough; had great thirst; difficult deglutition; tongue brown, dry and shrivelled; skin hot and moist; pulse quick and soft; bowels constipated. The internal fauces appeared red and inflamed, and were covered with portions of a whitish membranous film: but no swelling or enlargement of the tonsils, uvula, or velum pendulum palati.

Reviewing the whole train of symptoms, and observing the great anxiety, difficulty of breathing, and peculiar sound of respiration, I had no hesitation in pronouncing his complaint to be Cynanche Trachealis.

I directed a large bleeding from the arm: the gluten appeared separated, and lying on the surface of the crassamentum, as in inflammatory diseases. Prescribed one grain of tartarized antimony every half hour.—In this manner his medical attendant (who remained with him the whole time) gave him eight grains of the antimonium tartarizatum, which produced produced considerable nausea, some vomiting, and a copious evacuation from the bowels. Fifty drops of laudanum were now given him, and a large blister applied to the throat, and another to the nape of the neck.

29th.—Visiting him this morning, I understood that he had slept a little, subject to frequent interruptions, as the difficulty of breathing increased by paroxysms, with great restlessness and anxiety, seemingly threatening immediate suffocation. With each fit of coughing he had brought up some mucus of a whitish appearance, resembling coagulated lymph; but the efforts to bring it up were very distressing.

He sat up, and his attendants believed him better; but the affection of the system was more considerable, with a weak, quick pulse, beating 120 in a minute, skin warm and relaxed. The shrill cough and croaking noise continued with increasing severity. With a view to promote expectoration, and if possible, dislodge the matter which was evidently accumulating in the trachea, I directed a continuance of the tartarized antimony; and a grain was given him every hour. About four grains in the whole was taken, but did not excite vomiting; and any increase of expectoration was unexpected in so short a time.

I now directed him to take a spoonful of a decoction of seneka (℥j ad ℔j) as often as possible; of this, I believe, but little was swallowed; and, as in the case of General Washington, speaking, which was painful from the beginning, now became scarcely practicable. About four o'clock, A. M. he grew worse, breathed with more and more difficulty, and expired a few minutes before five; being thirty hours after my first visit.

I regret my not being permitted to open the body. Country situations are, in this respect, unfavourable to medical practice.

I am, &c.

ROBERT BEAVER, M. D.

*Carnarvon, North Wales,*
*May* 15, 1811.

